# Mediterranean Diet in Older Irish Adults: Prevalence, Patterns, Predictors and Pertinence

**DOI:** 10.3390/nu16162615

**Published:** 2024-08-08

**Authors:** Catherine Norton, Elaine Clarke, Pablo J. Marcos-Pardo, Audrey Tierney

**Affiliations:** 1 Department of Physical Education & Sport Sciences, Faculty of Education and Health Sciences, University of Limerick, V94 T9PX Limerick, Ireland; elaine.clarke@mail.itsligo.ie; 2 Health Research Institute, University of Limerick, V94 T9PX Limerick, Ireland; audrey.tierney@ul.ie; 3 Department of Health and Nutritional Science, Institute of Technology Sligo, F91 YW50 Sligo, Ireland; 4 Department of Education, Faculty of Education Sciences, University of Almería, Carretera Sacramento s/n, La Cañada de San Urbano, 04120 Almería, Spain; pjmarcos@ual.es; 5 SPORT Research Group (CTS-1024), CERNEP Research Center, University of Almería, Carretera Sacramento s/n, La Cañada de San Urbano, 04120 Almería, Spain; 6 Discipline of Dietetics, School of Allied Health, Faculty of Education and Health Sciences, University of Limerick, V94 T9PX Limerick, Ireland; 7 Department of Dietetics, Nutrition and Sport, La Trobe University, Melbourne, VIC 3086, Australia

**Keywords:** nutrition, mediterranean diet, chronic diseases, non-communicable diseases

## Abstract

The Mediterranean diet (MD) shows the strongest evidence in support of healthy aging and prevention of age-related diseases. It is associated with a decreased risk of chronic disease, such as cardiovascular disease, obesity, hypertension, diabetes mellitus and cognitive disease. Given the health-promoting aspects of this diet, we conducted a secondary analysis of data from the LifeAge study (“Promoting the shift: sedentary Lifestyle towards active Ageing-LifeAge” Project No 603121-EPP-1-2018-1-ES-SPO-SCP) with the primary aim to determine the proportion of older Irish adults adhering to the MD and to examine the association between adherence to the MD (assessed using the Mediterranean Diet Adherence Screener (MEDAS) scoring tool) and anthropometric risk factors of non-communicable diseases. Of the 131 eligible participants (71.8% female, n = 94) (medically well, aged > 50 years and physically independent) (mean age = 66.2 ± 6.5 years), the mean cumulative MD score across the cohort was 5.8 ± 2.2, with 41% classified as moderate-to-high MD adherers (scoring > 7 MEDAS). Females had a significantly higher score compared to males (female 6.24 ± 1.98; male 4.86 ± 2.53; *p* = 0.002). Age (y), waist circumference (cm) and waist–hip ratio (WHR) each had a significant (*p* < 0.05) weak and negative correlation with MD score (r = −0.193, −0.240, −0.284, respectively). Visceral fat level had a significant (*p* < 0.05) moderate and negative correlation with MD score (r = −0.327). This analysis is the first to assess adherence to the MD in older Irish adults. MD adherence was lower in the Irish older cohort than that reported in older adults in Mediterranean regions and was adhered to more by females and those with a more favourable anthropometric profile. The health-promoting aspects of the diet are evident in non-Mediterranean regions as is demonstrated by the lesser incidence of some non-communicable diseases in moderate-to-high adherers. With the evidence of the protective effects of the MD, external to Mediterranean regions, it would be helpful to establish limitations to adherence, especially in aging populations living in non-Mediterranean regions.

## 1. Introduction

The Mediterranean Diet (MD) describes the eating pattern traditionally followed by countries surrounding the Mediterranean Sea. The diet is largely plant-based with a high consumption of fruits, vegetables, legumes and complex carbohydrates, moderate consumption of fish, use of olive oil as the main culinary fat, low dairy and meat and moderate consumption of red wine with meals [1]. Traditions of the people in this region include preparing their own food, sharing meals with family and friends, being social and having a positive outlook, which may also contribute to the physical and mental health benefits associated with the lifestyle [2]. The MD is shown to be preventative against developing metabolic syndrome [3], cardiovascular disease (CVD) [4], inflammation [5,6], cancer [7], neurological disorders [8] and protecting cognitive health [9,10]. Adherence to the MD can have a positive impact on the mental health of older adults and may contribute to protecting geriatric patients with multimorbidity from developing depressive symptoms [11]. This diet shows the strongest evidence in support of healthy aging and prevention of diseases related to aging [12]. Consistent observance of the MD has shown an 8–10% reduction in mortality and death as a result of CVD [13] and furthermore is associated with higher cognitive function [14], reduced age-related cognitive decline [15,16] and maintenance of telomere length, which has been associated with age-related diseases and can be indicative of lifespan [17]. Among older adults, adherence to the MD combined with healthy lifestyle behaviours is associated with a 50% lower all-cause and cause-specific mortality rate [18].

Adherence to the MD can be assessed using the 14-item Mediterranean diet adherence screener (MEDAS) [19]. This dietary screening tool scores adherence to the MD by assessment of quantity and frequency of consumption of foods associated with the MD.

The older Irish adult population is set to increase significantly [20], concomitant with global populations. As of the last census, 13.4% of the population was aged over 65 years [21]; this figure is predicted to increase to 22.4% by 2041 [22]. Older Irish adults adhere poorly to Irish dietary guidelines [23], falling below the recommendations for fruit, vegetables, dairy, meat and other protein sources while overconsuming foods high in fat, sugar and salt [24]. While the benefits of a MD in areas outside of the Mediterranean region are not well established, research shows beneficial effects of the diet can be reproduced in other parts of the world [25].

Therefore, our aim was to determine the habitual adherence of older Irish adults to the MD and to examine the association between adherence to the MD and anthropometric risk factors for non-communicable diseases. Furthermore, we aimed to identify predictors of high adherence to a MD in a non-Mediterranean region.

## 2. Materials and Methods

### 2.1. Study Design

This study was conducted as part of the European Union Erasmus+ funded LifeAge project (“Promoting the shift sedentary Lifestyle towards active Ageing-LifeAge” Project No 603121-EPP-1-2018-1-ES-SPO-SCP), as detailed comprehensively elsewhere [26]. The study was conducted as a collaborative partnership between five European countries. Data presented here are specific to Irish participants only, and were collected at the University of Limerick, Ireland.

### 2.2. Participants

A convenience sample of community-dwelling adults was invited to participate. Participants (150) were recruited through advertisements in social and senior centres, and presentations given at communities close to the university. Inclusion criteria were (a) over 50 years old, and (b) being physically independent. Exclusion criteria were (a) presence of musculoskeletal injuries or physical limitation that may have influenced test battery participation, (b) habitual use of medications known to influence physical test battery performance and (c) moderate to severe cognitive impairment diagnosed by a physician, or severe psychiatric problems. Ultimately, 127 Irish participants were included in data analysis for Life Age. Failure to present for testing (9), lack of consent (2), missing data points (11), and withdrawal (1) were among reasons for exclusion. However, 131 successfully completed the tests included in this current research and were included in our analysis.

### 2.3. Ethics

In compliance with the Declaration of Helsinki in the conduct of human research, all procedures were undertaken following approval of the Faculty of Education and Health Sciences Research Ethics Committee of the University of Limerick (2018_02_15_EHS) and participants’ written, informed consent was obtained.

### 2.4. Procedures

Following appropriate screening and provision of written informed consent, participants presented to the University of Limerick for comprehensive assessment of sociodemographic, anthropometric, dietary, and physical and mental wellness variables. This assessment was completed by a team of researchers over approximately 3 h on the same day. The order of assessment was maintained for all participants.

### 2.5. Variables and Instruments

Body mass (kg) was measured to the nearest 0.1 kg on an electronic scale (Tanita BC-418, Arlington Heights, IL, USA) with the participant in light clothes and barefoot. Height (cm) was measured to the nearest millimetre using a stadiometer (Tanita HR001, Arlington Heights, IL, USA). Body mass index (BMI) (kg/m^2^) was calculated by dividing weight by height^2^ with BMI categories classified as underweight < 18.5 kg/m^2^, normal weight 18.5–24.9 kg/m^2^, overweight 25–29.9 kg/m^2^ and obese > 30 kg/m^2^ [27]. The International Society for Advancement on Kinanthropometry (ISAK) guidelines were followed for measuring the kinanthropometric parameters [28] and completed by accredited (Level 1) practitioners. Body composition was measured by bioelectrical impedance analysis (BIA) scale (Tanita BC-418, Arlington Heights, IL, USA).

Adherence to the MD was assessed using the Mediterranean Diet Adherence Screener (MEDAS) scoring tool [19]. This instrument comprises 14 questions regarding the main groups of food consumed as part of the MD and is indicated to be a moderate and valid tool for the estimation of MD adherence [29]. Those scoring 7 or more were classified as moderate-to-high adherers and those scoring below 7 were classified as low adherers [26].

Participants were asked to indicate the presence of a chronic or long-term (>6 months) illness or health problem at screening, using the World Health Organization (WHO, 2020), chronic illnesses definition: “diseases of long duration and generally slow progression” and often include conditions such as heart disease, stroke, cancer, chronic respiratory diseases, and diabetes. Participants also reported habitual/chronic prescriptive medication use.

### 2.6. Statistical Analysis

Statistical analysis was completed using IBM SPSS statistics Version 26 (IBM Corp., New York, NY, USA). Continuous variables were assessed for normality using the Kolmogorov–Smirnov test. Demographic variables are reported by frequency (n, %) and mean ± standard deviation. The cohort was grouped into different age ranges; ‘middle-aged’ (<65 years old), ‘young old’ (65–74 years old), ‘old old’ (75–84 years old) and ‘oldest old’ (>85 years old) [30], BMI categories, adherence to the MD groups and gender. Independent samples *t*-tests, one-way ANOVA and Chi-square tests were used to analyse differences between continuous and categorical variables, respectively across groupings. Non-parametric alternatives were used for variables that were skewed. Bivariate correlation analyses were used to determine the strength and direction of the relationship between MD score and anthropometric, visceral fat, measures of physical activity and well-being variables. Depending on whether each variable was parametric or non-parametric, Pearson and Spearman’s rho bivariate correlation analyses were performed accordingly. R-values were reported for strength and direction of the relationship and *p*-values represent significance of the relationship. R-values were classified as weak (0.10 to 0.29), moderate (0.30 to 0.49) or strong (0.50 to 1.00) correlations. Logistic regression analysis was performed to determine which variables were associated with moderate-to-high MD adherence. Significant differences between low and moderate-to-high adherence at univariate level (*p* < 0.05) together with variables with significant correlation (*p* < 0.200) were included in multivariate models to assess independent predictors of moderate-to-high adherence, reported as odds ratio (OR) with 95% confidence interval (CI).

## 3. Results

A total of 131 eligible participants were recruited. Table 1 presents demographic details for a predominantly female cohort (71.8%) with an average age of 66.2 ± 6.5 years with the majority (41.8%) categorised as ‘young old’ (83). There was a significant difference in the distribution of males and females across the age categories where more men were represented in the ‘middle-aged’ (male 40.5%, female 36.2%), and ‘old old’ (male 29.7%, female 5.3%) categories and more women in the ‘young old’ (female 55.3%, male 29.7%), and ‘oldest old’ (female 3.2%, male 0%) groups (*p* < 0.0005). The mean BMI was 26.8 ± 5.1 kg/m^2^ with a significant difference between males and females (males 28.9 ± 3.9 kg/m^2^, females 26.2 ± 4.9 kg/m^2^, *p* = 0.004). Nearly two-thirds of participants were classified as overweight (40%) or obese (23.8%). Within gender, more males were classified as overweight and obese, whereas more females were classified as normal weight and overweight (*p* = 0.002). Of the cohort, 65.6% were married or remarried and 69.8% were living with their spouse; 70.2% were retired and 40.3% attained a degree or higher.

Table 2 shows the cohort’s anthropometrics, health status and habitual prescriptive medication use. Males had significantly higher visceral fat levels (male 15.9 ± 3.6; female 9.1 ± 2.5; *p* < 0.0005), waist circumference (cm) (male 100.8 ± 9.9; female 86.6 ± 14.6; *p* < 0.0005) and waist–hip ratio (WHR) (male 1.0 ± 0.1; female 0.8 ± 0.1; *p* < 0.0005). Of the total cohort, 46.3% reported a chronic health problem. Thirty-four percent (34%) of participants reported using anti-hypertensive medications and 38.2% using statins. Males were prescribed and taking significantly more antihypertensive medications (males = 48.6%, females = 28.7%, *p* = 0.031) and statins (males = 64.9%, females = 27.7%, *p* < 0.0005), with nutritional supplements reportedly taken significantly more by females (females = 24.5%, males = 5.4%, *p* = 0.012). Across BMI groups there was a statistically significant difference in those using statins where most of the use was in overweight participants (55.8%) compared to obese (25.8%), normal weight (25.6%) and underweight (25%) participants (*p* = 0.007).

The mean cumulative MD score for the cohort was 5.8 ± 2.2. Females had a significantly higher MD score compared to males (females 6.2 ± 1.9; males 4.8 ± 2.5; *p* = 0.002). Table 3 presents the group mean intake of the individual components of the MD assessed using the MEDAS scoring tool in comparison to the MD recommendations [19]. The cohort met the MD criteria, as referred to in Table 3, for recommendations of servings of vegetables per day (2.1 ± 1.2), servings of red meat, hamburger or meat products per day (0.78 ± 0.6), number of sweet or carbonated beverages per day (0.2 ± 0.6) and number of dishes with vegetables, pasta, rice or other seasoned with sofrito per week (2.4 ± 2.3). Key MD criteria including consumption of olive oil, fruit, nuts, legumes, fish and wine were below recommendations, while consumption of commercial sweets and pastries, butter, margarine or cream were above recommendations. A significantly higher percentage of females met the recommendations for servings of vegetables per day (*p* = 0.007), fruit per day (*p* = 0.001), servings of red meat, hamburger or meat products per day (*p* = 0.042), number of sweet or carbonated beverages per day (*p* = 0.027) and preferential consumption of chicken, turkey or rabbit instead of veal, pork, hamburger or sausage (*p* = 0.036) compared to males.

Low MD adherence was defined as having a MD cumulative score of less than seven and moderate-to-high adherence was defined as having a cumulative score of seven or more [26]. The mean MD score of the low adherers was 4.3 ± 1.4 compared to the moderate-to-high adherers 8.1 ± 1.0 (*p* < 0.0005). There was a significant difference in the representation of males and females in the MD adherence categories. More females (46.8%) were moderate-to-high adherers compared to males (27%) (*p* = 0.038). Low adherers were significantly older (67.1 ± 6.6 years old) compared to the moderate-to-high adherers (65 ± 6.4 years old) (*p* = 0.042). There was a significant difference in BMI, whereby low adherers had a significantly greater BMI (27.7 ± 4.8 kg/m^2^) compared to moderate-to-high adherers (25.8 ± 4.7 kg/m^2^) (*p* = 0.025).

We compared gender, age, MD score, health status and medication use between low adherers to moderate-to-high MD adherers (Table 4). Across MD adherence groups, low adherers had a significantly higher visceral fat level (low adherers 12 ± 4.4; moderate-to-high adherers 9.6 ± 3.4; *p* = 0.001) and waist circumference (cm) (low adherers 93.3 ± 14.1; moderate-to-high adherers 86.6 ± 15.2; *p* = 0.018). Moderate-to-high adherers had a significantly lower WHR (moderate-to-high adherers 0.8 ± 0.1; low adherers 0.9 ± 0.1; *p* = 0.040). The use of nutritional supplements was significantly higher in the moderate-to-high adherence MD group (27.8%) compared to low adherers (13%) (*p* = 0.034). Although a not significant 52.7% of the low adherers to the MD indicated a chronic health problem, 40.3% use anti-hypertensive medications and 41.6% use statins compared to moderate-to-high adherers (37.3%, 25.9%, 33.3%, respectively). 

Age, waist circumference and WHR each had a significant (*p* < 0.05) weak and negative correlation with MD score (r = −0.193, −0.240, −0.284, respectively). Visceral fat level had a significant (*p* < 0.05) moderate and negative correlation with MD score (r = −0.327).

Variables that were significantly different at the univariate level (*p* < 0.05), variables with significant (*p* < 0.200) correlation with MD score, together with age, BMI and demographic variables (education attainment, occupational status, marital status, living arrangements) were entered into a multivariate model to assess independent predictors of being in a moderate-to-high MD adherence group (Table 5). The model as a whole explained 10.9% (Cox and Snell R square) and 14.7% (Nagelkerke R squared) of the variance in adherence, and correctly classified 66.6% of cases. Visceral fat was the strongest predictor of moderate-to-high adherence to the MD, whereby increased visceral fat negatively predicts moderate-to-high MD adherence (B = −0.209, *p* = 0.006, 95% CI 0.699, 0.941).

## 4. Discussion

The aim of this study was to investigate older Irish adults’ adherence to the MD, to examine the association between adherence to the MD and risk factors for non-communicable diseases and to identify predictors of high adherence to a MD in a non-Mediterranean region. To the author’s knowledge, this is the first study to investigate habitual adherence to a MD in a cohort of Irish older adults. The study found that in this cohort of medically well, aging adults, there was overall low adherence to the MD and key components of the diet such as intakes of olive oil, fruit, nuts, legumes and fish were not achieved. More females adhered to the MD principles than males. Participants who were low adherers tended to be older with a higher BMI, visceral fat level, waist circumference and WHR compared to those who moderately or highly adhered to the MD.

The average waist circumference indicated central obesity, which may increase the risk of CVD [31], with the average WHR for males indicative of metabolic risk [32]. Males had a higher visceral fat level, which is associated with the production of inflammatory mediators and associated with insulin resistance [33]. An unfavourable anthropometric profile is a risk factor for developing metabolic syndrome, CVD and other diseases and is also associated with disability and reduced quality of life. The health consequences resulting from excess weight in older adults have social and economic implications including increased healthcare costs [34]. Measuring central obesity, as opposed to BMI, has proven to be more effective at predicting risk to metabolic health and all-cause mortality [35]. When compared to those under 23 kg/m^2^, evidence suggests that for older adults within the overweight BMI category, there is no increased risk of mortality [36]. Therefore, meeting energy and nutrient targets through a high-quality diet to sustain and maintain a healthy weight in this cohort is paramount to healthy aging [37]. In addition, dietary patterns rich in fruits and vegetables, and protein-rich foods are positively associated, whereas dietary patterns high in sugar and fat are negatively associated with changes in healthy aging [37].

MD adherence was measured using the 14 item MEDAS tool, which has proven valid and reliable in several other non-Mediterranean regions [38,39,40,41]. The mean reported MD score was categorised as low adherence which may be due to the unfamiliarity of the MD in this age group particularly and reliance on key Irish dietary staples such as bread, meat and potatoes [42], which are not fundamental aspects of the MD. Similar to Irish national food consumption data, there was a low consumption of wine, sweet or carbonated beverages, oil, legumes, and nuts, and a high consumption of commercial sweets or pastries, fruit and butter with preferential consumption of white meat over red, in this cohort [42]. Among the older Irish population, there is poor adherence to Irish dietary guidelines [24], and as such, this finding not only represents low adherence to the MD but may indicate similar adherence to the national healthy eating guidelines.

When comparing the participants’ MD scores to similar cohorts in Mediterranean regions, differences are observed. In a study of Italian older adults, the average MD score was higher compared to this group (7.2 ± 1.7). Compared to a Spanish group from the PREDIMED study, Irish participants reported lower adherence to criteria including olive oil, red and processed meat, butter, margarine and cream, wine, fish, sweets and pastries and sofrito dishes consumption. The percentage of those meeting the criteria for fruit, sweet or carbonated beverages, legumes and nuts was similar between groups, while only vegetable and preferential white meat consumption was higher among Irish participants [43].

In comparison to non-Mediterranean regions, low MD adherence (score 2.3–3.5) was similarly observed in adults and older adults in Northern Ireland, as assessed by a nine-point MD score [44]. Similar findings are observed with participants of this study and the MD adherence of UK older adults in adherence to olive oil, nuts and preferential white meat consumption. There were differences in the groups as far less of this cohort met the criteria for red and processed meat, butter, margarine and cream, wine and fish, while the UK group adhered less to vegetables, fruit, sweet or carbonated beverages, legumes, sweets and pastries and sofrito [40]. There were also differences noted when compared to Australian studies of older adults where baseline results of an intervention group showed higher MD adherence (7.1) compared to the Irish cohort of this study. In another study, an Australian older adult cohort reported a MD score of 5.2; the Irish cohort had higher adherence to using olive oil, vegetables, fruit, red and processed meat, legumes, preferential white meat and sofrito consumption. There was similar adherence to quantities of olive oil and sweet or carbonated beverages, whereas the Australians had higher adherence in terms of wine, butter, cream and margarine, fish, sweets and pastries and nuts recommendations [45].

Within this Irish cohort, there is a reported lower adherence to the criteria for olive oil, red and processed meat, butter, cream and margarine, wine and fish when compared to other countries. This trend may be due to differences in culture and eating habits. Meat is a dietary staple in Ireland [42]. There is a higher consumption of pig, bovine or poultry meat over fish [46], and it is recommended in our national dietary guidelines to consume two portions of meat, poultry, fish, eggs, beans or nuts per day [23]. Spreads including butter and oils are one of the main contributors to fat in the Irish diet [42]. Low consumption of extra virgin olive oil is in agreement with Ireland’s healthy eating guidelines to use oil sparingly [23]. Wine consumption is aligned with Irish guidelines of <17 units per week for men and <11 units per week for women [42,47].

It is recommended by Irish healthy eating guidelines to include fish as a source of high-quality protein with at least one portion being oily fish [48]. Increased consumption of extra virgin olive oil and fish in accordance with the MD would meet the Irish recommendations by increasing intake of MUFA and PUFA fatty acids and protecting against heart disease [48,49].

Although not significant, moderate-to-high MD adherers had a lower prevalence of chronic disease, lower reported use of CVD medications and a generally more favourable anthropometric profile in comparison to those with low adherence. These results reflect evidence suggesting a reduced risk of metabolic disease [50], lower prevalence of chronic health problems [51] and the potential cardio-protective effects associated with MD adherence [52].

The CVD protective effect of the MD has been observed in a follow-up study of middle-aged and older US women where a reduction in risk factors including inflammation, hypertension, dyslipidaemia, BMI and insulin resistance yielded a reduced risk of cardiovascular events [53]. In a study of Caucasian older adults, MD adherence was shown to be protective of excess weight and increased visceral fat [54]. As much as a 26% reduced risk of hypertension has been observed with MD adherence [55]. Other favourable effects include improved serum lipid profiles of healthy individuals [56,57], where adherence to the MD resulted in reduced LDL cholesterol by increasing clearance of LDL cholesterol and limiting cholesterol absorption [58]. This has proven beneficial for those with a low-risk profile for CVD [56] and so the diet may be implemented as primary and secondary prevention of CVD.

The MD is rich in plants, unsaturated fat and polyphenols, which has shown to be a sustainable and effective method of CVD prevention [19]. Ireland’s older adult population may benefit from adopting this dietary pattern, which could potentially confer protective effects.

As observed in Spanish older adults, older age predicts greater MD adherence [59], which may be attainable for older adults in Mediterranean regions being more familiar with the traditional Mediterranean lifestyle including diet, physical activity and socializing [60]. In addition, a recent study indicates that in older adults at high CVD risk, increased physical activity levels and adherence to the MD for one year were associated with a lower inflammatory score, which was partly mediated by a reduction in body fat [61]. In other studies, higher education predicted adherence [10,43,62] possibly due to the relationship between higher education attainment and adherence to a healthy diet [63]. Higher education is associated with increased nutritional knowledge and being environmentally aware, which may encourage adherence to the MD due to its health and sustainability attributes [64]. In a study of Greek older adults, those with hypertension were less likely to adhere to the MD [55], and adherence to the MD was associated with a decrease in visceral adiposity proving the protective effect of central obesity [54]. In this study, similar findings were noted as visceral fat levels negatively predicted moderate-to-high MD adherence. Knowing which factors predict higher adherence to the MD in this group informs which cohort should be targeted to improve MD adherence.

While we observed an association between low adherence to the Mediterranean diet and increased anthropometric risk factors, the temporal sequence of these events remains unclear. Specifically, we cannot determine whether poor diet adherence led to the development of these risk factors or if pre-existing risk factors influenced dietary behaviours. This ambiguity introduces the possibility that the observed health outcomes may have caused changes in diet rather than the diet causing the health outcomes. As such, the potential for reverse causation cannot be excluded. To establish causal relationships more definitively, we recommend that future research employ prospective designs to better understand the temporal dynamics between diet adherence and health outcomes. Such studies could provide more robust evidence of causality and help to delineate the directionality of these relationships.

A limitation of this study is that the cohort recruited was not entirely representative of the older Irish adult population, and as such, the translation of the results needs to be interpreted cautiously. People who are motivated by health and wish to improve their lifestyle tend to come forward for related research participation [65], which may contribute to the cohort of this study being medically well and more physically active [66]. As such, higher use of nutritional supplements among moderate-to-high MD adherers may be explained by a health-conscious lifestyle [67].

A further potential limitation of our research is the possibility of residual confounding. Although we adjusted for several key covariates, such as age, gender, smoking status and physical activity, we acknowledge that unmeasured variables might still influence our findings. Residual confounding occurs when variables not included in the analysis affect the relationship between the exposure and the outcome. In our study, potential unmeasured confounders could include genetic predispositions, other lifestyle factors (e.g., stress levels, sleep quality), and socio-economic variables (e.g., income, education level). These factors could have a significant impact on both diet adherence and health outcomes, and their absence in our analysis may introduce bias. To mitigate these limitations, our future studies aim to collect comprehensive data on these and other potential confounders.

Other limitations include a lack of male participants. Additionally, the cross-sectional nature of the study and the assessment of MD adherence were not complemented with a three-day food diary or food frequency questionnaire to confirm the findings.

This study may inform future targeted dietary interventions in a similar cohort. From this study, practical implications for Irish dietary habits may include transitioning from foods high in saturated fats to foods containing monounsaturated and polyunsaturated fats such as reducing consumption of red meat and increasing portions of fish per week. This combined with liberal use of extra virgin olive oil and increased consumption of vegetables, legumes and nuts may aid in increasing adherence to the MD among the Irish adult population and may aid with a more favourable anthropometric profile.

With the known benefits of the MD, investigating barriers and enablers to the diet among the older Irish adult population warrants further research to inform future policies for promoting this diet.

## 5. Conclusions

We report for the first time, adherence to the MD in an older Irish adult cohort and identify potential predictors of high adherence. Adherence for the group was overall low, and although the cohort was somewhat representative of a more mid-to-high socioeconomic status, it highlighted that a lower visceral fat level was an independent predictor of moderate-to-high adherence to a MD. Better adherence to some key MD components may overlap with the national dietary guidelines. Investigating enablers and barriers in adhering to better diet quality in the aging population is warranted to prevent diet-related non-communicable diseases.

## Figures and Tables

**Table 1:** Demographic characteristics.

	Total n = 131 n (%)	Male n = 37 (28.2%) n (%)	Female n = 94 (71.8%) n (%)	*p* Value
Age (years) mean ± SD	66.3 ± 6.5	67.4 ± 8.3	65.8 ± 5.7	0.207
Middle age > 65 years	49 (37.4)	15 (40.5)	34 (36.2)	
Young old 65–74 years	63 (48.1)	11 (29.7)	52 (55.3)	<0.0005
Old old 75–84 years	16 (12.2)	11 (29.7)	5 (5.3)	
Oldest old > 85 years	3 (2.3)	0 (0)	3 (3.2)	
BMI (kg/m^2^) mean ± SD	26.8 ± 5.1	28.9 ± 3.9	26.2 ± 4.9	0.004
Underweight < 18.5 kg/m^2^	4 (3.1)	0 (0)	4 (4.3)	
Normal weight 18.5–24.9 kg/m^2^	43 (33.1)	4 (10.8)	39 (41.9)	0.002
Overweight 25–29.9 kg/m^2^	52 (40.0)	21 (56.8)	31 (33.3)	
Obese > 30 kg/m^2^	31 (23.8)	12 (32.4)	19 (20.4)	
Marital Status				
Single, never married	15 (11.5)	2 (5.4)	13 (13.8)	
Married, first and only marriage/Remarried	86 (65.6)	28 (75.7)	58 (61.7)	0.161
Separated/divorced	14 (10.7)	5 (13.5)	9 (9.6)	
Widowed	16 (12.2)	2 (5.4)	14 (14.9)	
Occupational Status				
Full-time worker	16 (12.2)	11 (29.7)	5 (5.3)	
Part-time worker	18 (13.7)	1 (2.7)	17 (18.1)	
Unemployed	2 (1.5)	0 (0)	2 (2.1)	0.001
Self-employed	3 (2.3)	1 (2.7)	2 (2.1)	
Retired	92 (70.2)	24 (64.9)	68 (72.3)	
Education level attained				
No qualification	2 (1.6)	1 (2.7)	1 (1.1)	
Second level	25 (19.4)	8 (21.6)	17 (18.1)	
Less than a higher education	15 (11.6)	4 (10.8)	11 (11.7)	0.354
Higher education below degree	35 (27.1)	5 (13.5)	30 (31.9)	
Degree or higher	52 (40.3)	19 (48.6)	34 (36.2)	
Living Arrangements n				
Living with spouse	90 (69.8)	29 (78.4)	61 (65.6)	
Cohabiting with partner	6 (4.7)	3 (8.1)	3 (3.2)	0.032
Living alone	33 (25.6)	4 (10.8)	29 (31.2)	

SD = standard deviation, BMI = body mass index. *p* value derived with independent sample *t*-test for normal continuous variables, Mann–Whitney test for non-parametric continuous variables and Chi-square test for categorical variables, significant at *p* < 0.05.

**Table 2:** Anthropometrics, health status and habitual prescriptive medication use.

	Total n (%)	Male n = 37 (28.2%) n (%)	Female n = 94 (71.8%) n (%)	*p* Value
BMI (kg/m^2^) (Mean ± SD)	26.9 ± 4.8	28.8 ± 3.9	26.2 ± 4.9	0.004
Visceral fat (level) (Mean ± SD)	11.0 ± 4.2	15.9 ± 3.6	9.1 ± 2.5	<0.0005
Waist Circumference (cm) (Mean ± SD)	90.6 ± 14.9	100.8 ± 9.9	86.6 ± 14.6	<0.0005
Hip Circumference (cm) (Mean ± SD)	107.0 ± 43.8	104.3 ± 11.1	103.1 ± 10.5	0.553
Waist: Hip Ratio (WHR) (Mean ± SD)	0.9 ± 0.1	1.0 ± 0.1	0.8 ± 0.1	<0.0005
>600 METs/week	107 (81.7)	28 (75.7)	79 (84.0)	0.317
Chronic health problem indicated	57 (46.3)	16 (43.2)	41 (47.7)	0.550
Reported use of anti-hypertensive medication	45 (34.4)	19 (48.6)	27 (28.7)	0.031
Reported use of statins	50 (38.2)	24 (64.9)	26 (27.7)	<0.0005
Reported use of nutritional supplement	25 (19.1)	2 (5.4)	23 (24.5)	0.012
Reported use of Vitamin D supplement	7 (5.3)	0 (0)	7 (7.4)	0.190

SD = standard deviation, BMI = body mass index. *p* value derived with independent sample *t*-test for normal continuous variables, Mann–Whitney test for non-parametric continuous variables and Chi-square test for categorical variables, significant at *p* < 0.05.

**Table 3:** Mean intake of Mediterranean Diet components as per the MEDAS checklist and the percentage of participants that met the recommendations.

Mediterranean Diet Criteria [19]	Servings Mean ± SD	Total n (%)	Male (n = 37) (28.2%) n (%)	Female (n = 94) (71.8%) n (%)	*p* Value
Low adherence (<7 score)		77 (58.5)	27 (73)	50 (53.2)	
Moderate-to-high adherence (>7 score)		54 (41.2)	10 (27)	44 (46.8)	0.038
Use of olive oil as main culinary fat	N/A	77 (58.8)	20 (54.1)	57 (60.6)	0.491
≥4 tbsp of olive oil/day	1.1 ± 1.6	5 (3.8)	1 (2.7)	4 (4.3)	1.000
≥2 servings (serving: 200 g) of vegetables/day	2.1 ± 1.2	84 (64.1)	17 (45.9)	67 (71.3)	0.007
≥3 fruit units/day	2.8 ± 1.6	67 (51.1)	10 (27)	57 (60.6)	0.001
<1 serving (serving: 100–150 g) of red meat, hamburger, or meat products/day	0.8 ± 0.6	46 (35.1)	8 (21.6)	38 (40.4)	0.042
<1 serving (serving: 12 g) of butter, margarine or cream/day	1.6 ± 2.4	25 (19.1)	6 (16.2)	19 (20.2)	0.600
<1 sweet or carbonated beverages/day	0.2 ± 0.6	113 (86.3)	28 (75.7)	85 (90.4)	0.027
≥7 glasses of wine/week	1.9 ± 1.3	9 (6.9)	2 (5.4)	7 (7.4)	1.000
≥3 servings (serving: 150 g) of legumes/week	2.1 ± 2.2	39 (29.8)	12 (32.4)	27 (28.7)	0.676
≥3 servings of fish (serving: 100–150 g or 4–5 units) or shellfish (200 g/week)	1.9 ± 1.3	33 (25.2)	6 (16.2)	27 (28.7)	0.138
Consume commercial sweet or pastries < 3 times/week	3.6 ± 4.5	47 (35.9)	13 (35.1)	34 (36.2)	0.911
≥3 servings (serving: 30 g) of nuts/week	2.4 ± 2.7	46 (35.1)	10 (27)	36 (38.3)	0.224
Preferentially consume chicken, turkey or rabbit instead of veal, pork, hamburger or sausage	N/A	104 (79.4)	25 (67.6)	79 (84)	0.036
Consume vegetables, pasta, rice or other dishes seasoned with sofrito ≥ 2 times/week	2.4 ± 2.3	72 (55)	22 (59.5)	50 (53.2)	0.516
Cumulative score (Mean ± SD)		5.8 ± 2.2	4.8 ± 2.5	6.2 ± 1.9	0.002

SD = standard deviation, N/A = not applicable. *p* value derived with independent sample *t*-test for normal continuous variables, Mann–Whitney test for non-parametric continuous variables and Chi-square test for categorical variables, significant at *p* < 0.05.

**Table 4:** Comparison of gender, age, MD score, health status and medication use between low adherers to moderate-to-high MD adherers.

	Low Adherers n = 77 (58.8%) n (%)	Moderate-to-High Adherers n = 54 (41.2%) n (%)	*p* Value
Males	27 (35.1)	10 (18.5)	
Females	50 (64.9)	44 (81.5)	0.002
Age	67.1 ± 6.6	65 ± 6.4	0.042
MD Cumulative Score (Mean ± SD)	4.3 ± 1.4	8.1 ± 1.0	<0.0005
BMI (kg/m^2^) (Mean ± SD)	27.4 ± 4.8	25.8 ± 4.6	0.025
Visceral fat (level) (Mean ± SD)	12 ± 4.4	9.6 ± 3.4	0.001
Waist Circumference (cm) (Mean ± SD)	93.3 ± 14.1	86.6 ± 15.2	0.018
Hip Circumference (cm) (Mean ± SD)	104.4 ± 10.9	101.8 ± 10.3	0.200
Waist: Hip Ratio (WHR) (Mean ± SD)	0.9 ± 0.1	0.8 ± 0.1	0.040
>600 METs/week	64 (83.1)	43 (79.6)	0.651
Chronic health problem indicated	38 (52.8)	19 (37.3)	0.235
Reported use of anti-hypertensive medication	31 (40.3)	14 (25.9)	0.089
Reported use of statins	32 (41.6)	18 (33.3)	0.340
Reported use of nutritional supplement	10 (13)	15 (27.8)	0.034
Reported use of Vitamin D supplement	4 (5.2)	3 (5.6)	1.000

MD = Mediterranean diet, SD = standard deviation, BMI = body mass index, METs = metabolic equivalents. *p* value derived with Chi-square test for categorical variables, independent *t*-test for normal continuous variables and Mann–Whitney test for non-parametric continuous variables significant at *p* < 0.05.

**Table 5:** Multivariate logistic regression analysis predicting moderate-to-high MD adherence.

Variable	Beta	OR (95% CI)	*p* Value
Age (years)	−0.023	0.98 (0.91, 1.04)	0.510
BMI category (overweight/obese = 0, normal weight = 1)	−0.614	0.54 (0.18, 1.58)	0.263
Visceral fat level	−0.209	0.81 (0.69, 0.94)	0.006
Education (lower = 0, higher = 1)	0.002	1.00 (0.43, 2.28)	0.997
Anti-hypertensive medications (using = 0, not using = 1)	−0.003	0.99 (0.41, 2.39)	0.995

## Data Availability

The original contributions presented in the study are included in the article, further inquiries can be directed to the corresponding author.
